# Shaped 3D Singular Spectrum Analysis for Quantifying Gene Expression, with Application to the Early Zebrafish Embryo

**DOI:** 10.1155/2015/986436

**Published:** 2015-10-01

**Authors:** Alex Shlemov, Nina Golyandina, David Holloway, Alexander Spirov

**Affiliations:** ^1^Faculty of Mathematics and Mechanics, St. Petersburg State University, Universitetsky Pr. 28, St. Peterhof, St. Petersburg 198504, Russia; ^2^Mathematics Department, British Columbia Institute of Technology, 3700 Willingdon Avenue, Burnaby, BC, Canada V5G 3H2; ^3^Computer Science and CEWIT, SUNY Stony Brook, 1500 Stony Brook Road, Stony Brook, NY 11794, USA; ^4^The Sechenov Institute of Evolutionary Physiology & Biochemistry, Torez Pr. 44, St. Petersburg 194223, Russia

## Abstract

Recent progress in microscopy technologies, biological markers, and automated processing methods is making possible the development of gene expression atlases at cellular-level resolution over whole embryos. Raw data on gene expression is usually very noisy. This noise comes from both experimental (technical/methodological) and true biological sources (from stochastic biochemical processes). In addition, the cells or nuclei being imaged are irregularly arranged in 3D space. This makes the processing, extraction, and study of expression signals and intrinsic biological noise a serious challenge for 3D data, requiring new computational approaches. Here, we present a new approach for studying gene expression in nuclei located in a thick layer around a spherical surface. The method includes depth equalization on the sphere, flattening, interpolation to a regular grid, pattern extraction by Shaped 3D singular spectrum analysis (SSA), and interpolation back to original nuclear positions. The approach is demonstrated on several examples of gene expression in the zebrafish egg (a model system in vertebrate development). The method is tested on several different data geometries (e.g., nuclear positions) and different forms of gene expression patterns. Fully 3D datasets for developmental gene expression are becoming increasingly available; we discuss the prospects of applying 3D-SSA to data processing and analysis in this growing field.

## 1. Introduction

Recent advances in microscopy technologies, biological markers, and automated processing methods are enabling sustained progress towards the long-standing goal of reconstructing embryogenesis by integrating cellular behavior and molecular dynamics [1–4]. New technology, including photonic microscopy approaches (reviewed in [5, 6]) and new biological markers (fluorescent proteins, photoactivatable compounds, and fluorescent nanoparticles such as quantum dots) [7], is producing quantitative high resolution data (spatial and temporal) at all levels of organization [8–12]. Reconstruction of a developmental atlas can be considered in stages, proceeding from an automated reconstruction of a cell lineage tree in space and time, annotated with quantitative information for cell shape, and adding on the spatiotemporal dynamics of gene expression in the cells [2, 4, 5].

There are some impressive examples of progress in this new research area on several model experimental animals, where the details of embryo development have been tracked from the fertilized egg to the several thousand cell embryonic stages, with cellular level resolution quantitative data (in which each cell in the embryo is described by its 3D spatial coordinates, its lineage, and the expression level of the key genes).

Such large-scale research projects require not only new high-throughput experimental approaches, but also new quantitative mathematical and computational approaches for the processing, analysis, and modeling of such extensive datasets [10, 12–17].

For reconstructing the spatiotemporal dynamics of gene expression, the raw data sets are stacks of confocal microscope scans of early embryos (fixed or live). Data is usually the intensity of fluorescent markers for either the mRNA or proteins encoded by the genes of interest. Extracting this data requires image segmentation to identify the signal for each cell (or nucleus). This produces text-files with the spatial coordinates, gene expression levels, and lineage history of each cell.

A major goal of this processing is to collect reliable quantitative data for the fitting and verification of modern computer dynamic and stochastic models of developmental gene regulation at single cell resolution.

Examples of publicly available high resolution quantitative datasets for several experimental animals include the roundworm “*C. elegans*” (Web resource “EPIC” [18]), the fruit fly “*D. melanogaster*” (“BID BDTNP” [2, 19]), and the zebrafish “*D. rerio*” (“BioEmergences” [4, 20]). Such data can be seen as a combination of biological signal, biological noise, and experimental noise. Use of the fluorescent data requires a clear separation of these components. For example, deterministic gene regulatory models should be corroborated solely against the biological trend component; stochastic models will also include biological noise components.

The data in cellular resolution 3D gene expression atlases typically has very high noise, with contributions from aspects such as the intrinsic gene expression noise observed in prokaryotes and eukaryotes [21–27] and the disorder in cellular/nuclear positions. New quantitative approaches are needed to separate the raw expression data into signal and noise components [28]. While some animals' embryos have simpler geometries, being relatively flat with spherical or ellipsoidal cell layers (like the early* Drosophila* fly embryo), many types of embryos have inherently spatially three-dimensional cell order, adding methodological difficulties with respect to specimen thickness and optical nontransparency. Such 3D challenges require new experimental and computational approaches [29, 30].

Different embryo geometries can produce different spatial characteristics on the gene expression data. For instance, in the early* Drosophila* embryo the data can be considered as patterns on an ellipsoidal surface. With an appropriate two-dimensional convolution (e.g., cylindrical projection) the data can be studied with 2D image processing techniques [31].

There are, however, datasets of recent experimental data which are truly 3D and cannot be properly transformed and analyzed in 2D. A prime example is expression data from early zebrafish embryos, where nuclei (cells) are in several irregular layers on the fish egg. Such data requires techniques and algorithms for directly processing 3D data. The irregular distribution of nuclei in layers presents an added challenge, since most quantitative methods operate on spatially regular data points.

To this end, this paper introduces a new method for processing irregular data points scattered in layers in the vicinity of an ellipsoidal (spheroidal) surface. We present a nonparametric method, which can address in a nonbiased way the arbitrary spatial distribution and unknown noise character of the expression data. In [31] we presented extensions of 2D singular spectrum analysis (2D-SSA) to analyze 2D and surface 3D datasets from* Drosophila* confocal scans. These extensions, circular and shaped 2D-SSA, were applied to gene expression patterns in the thin nuclear layer just under the surface of the embryo. We demonstrated how circular and shaped 2D-SSA can decompose the expression data into identifiable components (trend and noise), as well as separating signals from different genes.

In this paper, we extend SSA to irregular three-dimensional expression data (3D-SSA). For an initial application of the approach, we focus on dealing with spatial irregularity, using real zebrafish data for the spatial coordinates of nuclei and the spatial gene expression patterns from the “MatchIT” and “AtlasIT” packages [4] but using artificial functions (simple math functions and a smooth approximation of an intensity indicator) for the expression (fluorescence) intensities.

**Figure 1:**
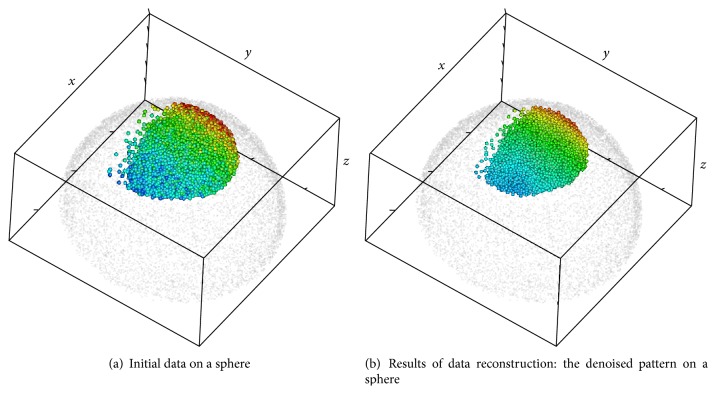
Zebrafish data [4] and the 3D-SSA approach: original nucleus position data for the spherical-cap gene expression region, with simulated intensity values (a schematic two-exponential pattern, with noise) represented by the color map; colors are given in the topographic scale, where the blue color corresponds to small values, when brown/red colors correspond to large values of intensity.

**Figure 2:**
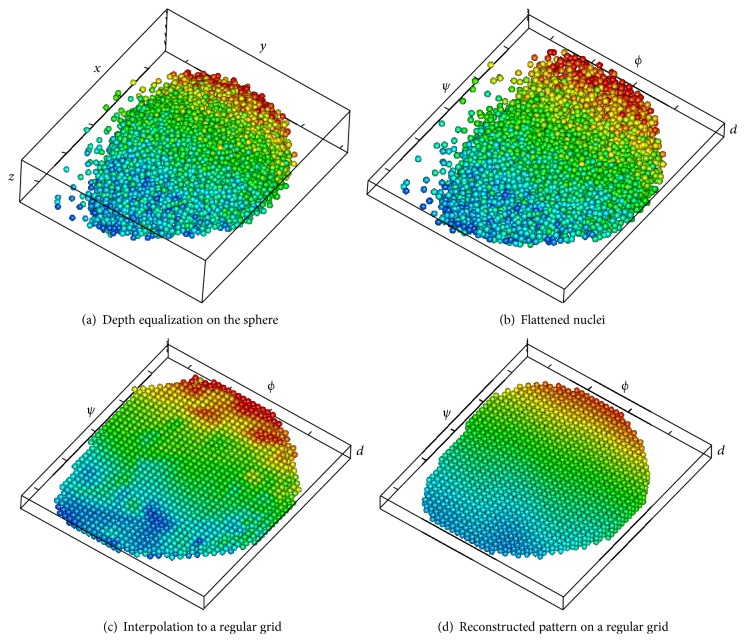
Processing steps in the 3D-SSA approach.

Figures 1 and 2 illustrate the main steps of the 3D-SSA approach (see details in Section 3).  Figure 1(a) shows simulated intensity data (a two-exponential pattern, with noise) on experimental data for nuclear positions in the spherical-cap geometry of the gene expression region.  Figure 2 shows the algorithm steps: depth equalization on the sphere, flattening, interpolation, and reconstruction.  Figure 1(b) shows the resulting pattern reconstruction after application of the 3D-SSA algorithm. Coloring of nuclei corresponds to gene expression intensities. Mixing of nuclei of different colors reflects noise. Regular (smooth) color patterns reflect noise removal.

This paper is structured as follows.  Section 2 describes the semiartificial datasets which were analyzed. The method is described in Section 3. In Sections 4 and 5 reliability of the approach is demonstrated on semiartificial data similar to real observations. Specifically, the first example considers all nuclei detected in the specimen at the shield stage, in which nuclei are distributed in a “spherical cap,” and expression (with noise) is generated for two patterns: (a) the sum of two exponentials and (b) bell-shaped.

The second example is for expression patterns similar to those for the nine regulatory genes characterized in [4]. Specifically, the test pattern is limited to nuclei where the* ntla* gene is found experimentally (the “MatchIT” package [4]). This set of nuclei is distributed in an equatorial strip. For its extraction we build a hull (envelope) of expressed nuclei (generally not convex since expressing area has complex shape). “Shaped” 3D-SSA is applied in all of the test cases.  Section 6 contains discussion and conclusions.

## 2. Data

In a 3D dataset from a zebrafish embryo, each datapoint corresponds to a nucleus, each represented by an array of numbers: three spatial coordinates for the nucleus centroid and the fluorescence intensities (in arbitrary units) of the labelled genes (usually two genes are labelled per embryo). Geometrically, the data points are distributed around a 3D ellipsoid in several irregular layers (see Figures 1, 4(a), 7(a), and 15(a)). If we approximate the fish egg as an ellipsoid (or spheroid), then the early fish embryo can be geometrically described as a thick (multilayer) spherical cap overlaying the egg, which can be flattened to a disc without substantial distortions at the margins (biologists refer to the geometry of these embryonic stages as “dome” or “disc”). The key genes studied at these early stages tend to form expression patterns in compact subareas of the spherical cap, such as open-ended rings and so forth. Preprocessing and SSA procedures can be focused or confined to these subareas. In other words, we assume that there is a transformation of a given expression area to a parallelepiped and that the transformation does not distort the data drastically. If the expression area is too large for such a transformation as a whole, then the transformation can be done as several independent pieces.

## 3. Method

We have adapted singular spectrum analysis (SSA) [32–34] to 2D spatial expression data [31] and shown it to be an efficient and robust means for data decomposition in these cases. The advantages of SSA, its adaptivity, flexibility with few parameters, visual control, and no prior specification of a noise model, make it promising for 3D analysis. A drawback of SSA its current lack of automation, though see [35] regarding automation on similar data structures.

SSA-type methods process data specified on a regular grid within a parallelepiped. Therefore, application to irregular 3D data first requires flattening, followed by regularization.

Processing 3D data which is in a layer near an ellipsoidal surface consists of the following steps:

(i) detection of data location: estimation of the ellipsoid center and finding the nuclear centroid positions relative to this, enabling data rotation for simpler, nondistorting flattening,
(ii) flattening the data and embedding them into a parallelepiped,
(iii) interpolation to a regular grid {*i* = 1,…, *N*_1_}×{*j* = 1,…, *N*_2_}×{*k* = 1,…, *N*_3_} to obtain *f*_*ijk*_,
(iv) application of 3D-SSA, perhaps by the shaped version to confine the analysis to subareas; 3D-SSA results in a decomposition of the form: *f*_*ijk*_ = *s*_*ijk*_ + *r*_*ijk*_, where *s*_*ijk*_ corresponds to the expression pattern or trend,
(v) interpolation of *s*_*ijk*_ back to regular nuclei on the parallelepiped,
(vi) transformation back to the original (irregular) embryo coordinates.

This process results in an extracted pattern with residual noise, on the original geometry. This allows for further study of the pattern's form (e.g., comparison with deterministic dynamic gene regulation models), as well as the model for the noise (e.g., to detect whether noise is additive or multiplicative).

Below, we comment on the steps of the processing scheme in further detail. For simplicity, we assume data are located near the surface of a sphere (the case for zebrafish eggs).

### 3.1. Detection of Data Location

The origin of the coordinate system is the center of the sphere, estimated as the point most equidistant from all data points (more formally, we find a point minimizing the variance of distances between its position and those of the data points).

Two types of spatial data distributions are considered, each of which has a specific procedure for reorientation and flattening.

The first type is for data located near the spherical cap. In this case, the “*z*”-axis passes through the center of the sphere and the middle point of the data; we can rotate the data to obtain positive “*z*”-values for all nuclear coordinates.

In the second type, data are located in a strip along the equator of the sphere. In this case, the “*z*”-axis is chosen orthogonal to the equatorial plane and passing through the center of the sphere.

In both cases, “*x*”- and “*y*”-axes are chosen orthogonal to the “*z*”-axis and to each other, oriented to maintain the original axis orientation as much as possible.

### 3.2. Flattening the Data

Data is embedded into a parallelepiped with sides parallel to the axes. The first axis corresponds to depth in the data layer, with the second and the third axes corresponding to surface directions. We aim to keep the proportions of the data as unchanged as possible.

#### 3.2.1. Depth Equalization of Data

We assume that the data are located in a layer of approximately constant depth on a spherical surface. Before projection, the data should be corrected to be within an ideal spherical layer of constant depth. Suppose that all nuclei are located in an area which is bounded by two convex surfaces (e.g., in a spherical layer).

The first step of the procedure is to find these surfaces as convex external and internal hulls (envelopes) applying the classical “convex hull” method to original data for finding the external hull and to inverted data to find the interior one. Then the found exterior and interior hulls are transformed to spherical surfaces of radii *R* + *D*/2 and *R* − *D*/2 correspondingly, where the sphere radius *R* is estimated as the median of distances from the data points to the sphere center, which was estimated on Section 3.1, and the layer depth *D* is estimated as the median distance between the hulls.

The procedure can detect a too-thin layer of one nucleus depth, when 2D-SSA should be used instead of 3D version. For data which is truly 2D, the 2D-SSA method discussed in [31, 36, 37] is applied. Otherwise, a new modified approach is elaborated here.

#### 3.2.2. Projection

Spherical projections can have different invariants. For data in spherical caps, we use the equidistant azimuthal projection [38, Section 25], to maintain distance from the center point (i.e., latitude) and therefore the original layer's linear sizes. After flattening and projection, the data points (nuclei) will therefore have the following coordinates: *ψ*, *φ* are coordinates along orthogonal meridians in the azimuthal projection and *d* represents depth; all are measured in metrical units.

For data in equatorial strips, we use the equidistant cylindrical projection [38, Section 12], maintaining distances of points to the equator (i.e., latitudes) and distances between points on the equator. This gives the similar coordinates *ψ*, *φ*, and *d* (latitude, longitude, and depth measured in metrical units). If an equatorial strip encircles with the whole equator, we obtain a parallelepiped with circular topology on the equatorial coordinate and can apply the circular version of SSA [39].

Note that we obtain new coordinates in approximately the same units (and the same proportions) as the original data. This is the main purpose of using equidistant projections, since proportions can strongly influence the interpolation to a regular grid.

We use relative coordinates for the flattened data. Thus in all pictures *d* is reported as a percentage, with 0% at the inner surface and 100% at the outer surface; *ψ*, *φ* are reported as fractions of the equator length.

### 3.3. Interpolation of Nuclei to a Regular Grid and Back

Interpolation of irregular data to a regular grid is known as a “3D scattered data interpolation problem” (see [40] for a description and an overview of different approaches).

We use a “triangulated irregular network-based linear interpolation using Delaunay triangulation” approach, where the interpolation is constructed by a linear interpolation of the vertex values from the corresponding triangulation simplex. Implementation is performed with the help of the library “CGAL” [41].

Note the nuclei do not necessarily pack the whole parallelepiped, and edge effects are a consideration. Therefore, after interpolation, we obtain the expression data on a subset of the parallelepiped grid. For cap-shaped data, for example, the subset is a disc. This subset can be processed with the shaped version of 3D-SSA.

For back-interpolation to nuclei, the conventional trilinear interpolation is used for pattern reconstruction. Residual values are calculated as differences between original and extracted-pattern (trend) expression values.

### 3.4. Shaped 3D-SSA

Shaped 3D-SSA methods need original data to be given on a subset of a regular grid (which we call the initial shape).

A parameter of the method is the shaped 3D window, which is inscribed in a parallelepiped of sizes *L*_1_ × *L*_2_ × *L*_3_.

It is assumed that the chosen window covers all the points of the original shape. If not, the uncovered grid points are removed. For a proper window shape, the number of removed points should not be large. If so, another window shape or size should be chosen.

Shaped 3D-SSA can be considered as a particular case of shaped SSA (see e.g., [37, 39]). To outline the algorithm, we do the following.

*Algorithm of Shaped 3D-SSA.*

(1) We will consider all possible locations of the window within the original data array and denote their number as *K*. For each location of the window we unfold the shaped window into a vector of length *L*. The obtained vectors are put together into a so-called trajectory matrix.
 The trajectory matrix **X** has a certain structure; since the windows cover the array points several times, a lot of matrix elements coincide. Such matrices are called quasi-Hankel [42] and form a linear space. We will designate this space *ℋ*.
 Define the* embedding operator 𝒯*(*𝕏*): *𝒳* → *ℋ*. This operator is linear, is an injection (if each point of the initial shaped array is covered by windows), and therefore is invertible.
(2) Construct the singular value decomposition (SVD) of the trajectory matrix **X**: **X** = ∑_*i*=1_^*d*^ *σ*_*i*_ *U*_*i*_ *V*_*i*_^T^. The eigenvectors *U*_*i*_ can be folded to eigenarrays, which have the same shape as the window has.
(3) Choose a subset *I* ⊂ {1,…, *d*} and put **X**_*I*_ = ∑_*i*∈*I*_ **X**_*i*_, where **X**_*i*_ = *σ*_*i*_ *U*_*i*_ *V*_*i*_^T^. Conventionally, the choice is *I* = {1,…, *r*}, *r* < *d*, what corresponds to an approximation of **X** by a low-rank matrix **X**_*I*_.
(4) Project the matrix **X**_*I*_ to the space *ℋ* and obtain the reconstructed image (pattern) as *𝕏*_*I*_ = *𝒯* ^−1^Π_*ℋ*_ **X**_*I*_.

Note that, by additivity, *𝕏*_*I*_ = ∑_*i*∈*I*_ *𝕏*_*i*_, where *𝕏*_*i*_ = *𝒯* ^−1^Π_*ℋ*_ **X**_*i*_ are called elementary components. Therefore the forms of elementary components can be used for detection of pattern components.

### 3.5. Choice of Parameters

Decomposition of gene expression data on irregular nuclear positions has several parameters: for interpolation and flattening; and for SSA (the window shape and size, and the number *r* of components for reconstruction).

In order to obtain a sufficient number of grid points with respect to the number of nuclei, steps of the regular grid should not be too large. The upper bound for the grid points is limited only by computational costs.

Recommendations for 3D-SSA are similar to that for 2D-SSA and 1D-SSA given in [33, 34, 37]. Larger windows correspond to more refined decomposition and more accurate reconstruction if the signal (pattern) has a simple structure generating a few SVD components. For more complex patterns, medium to small windows are preferable.

Note that window size is measured with respect to pattern features and should not depend on interpolation step. Therefore, window sizes are measured as a percentage of image sizes, not in the number of grid points. Starting window sizes can be chosen as approximately 10–20% of the image sizes in each direction. If the pattern is extracted imprecisely, the window can be enlarged; if the pattern is mixed with the residual (noise), the window should be decreased.

Identification of pattern components can be performed by analyzing the forms of eigenarrays or elementary reconstructed components (see [37]). Slowly varying patterns can be constructed readily by accumulation of slowly varying elementary components. Since it is difficult to perform a visual analysis of 3D objects, it is preferable to depict slices (1D graphs or 2D images), obtained by fixing one or two coordinates.

Quality of pattern extraction can be checked by means of residual behaviour. For proper pattern extraction, residuals should vary around zero. Thus it is recommended to choose slowly varying elementary components such that the residual has no part in the pattern.

### 3.6. Model of Residuals

For understanding biological sources of noise in gene expression, it is of interest to extract from the data a model of the noise (functional relation of the leading moments of gene expressions). We suggest a method for model detection based on a standard test of heteroscedasticity of residuals with different normalizations.

For a decomposition of initial data into pattern and noise: *x*_*i*_ = *s*_*i*_ + *r*_*i*_, *i* = 1,…, *N*, where *N* is the number of nuclei (enumeration by one index instead of three does not affect the results), assume that noise in nuclei is independent and consider the model

(1) $$\begin{array}{c} r_{i}=\varepsilon_{i}\cdot {\left|\;s_{i}\;\right|}^{\alpha }, \end{array}$$

where *Eε*_*i*_ = 0 and *Dε*_*i*_ = const.

If *α* = 0, the noise is additive (its standard deviation does not depend on pattern values). If *α* = 1, the noise is multiplicative (its standard deviation is proportional to pattern values). The intermediate value *α* = 0.5 corresponds to Poissonian noise, where noise variance is proportional to pattern value.

The Park method [43] estimates *α* as the slope of a linear regression in the model

(2) $$\begin{array}{c} \log\left|\;r_{i}\;\right|=\log\sigma +\alpha \log\left|\;s_{i}\;\right|+\nu_{i}, \end{array}$$

where *ν*_*i*_ = log|*ε*_*i*_/*σ*| is a well behaved error term. The Park method appears to be robust to the distribution of the residuals. An estimate of *α* in model (2) can be obtained, for example, by the least-squares method.

### 3.7. Implementation

We implemented all of the described methods in R [44] and included them in our BioSSA package.

For construction of convex and nonconvex hulls (the “alphashape” [45] method is used), the library “qhull” [46] is used by the wrapping R-packages (alphashape3d [47], geometry [48]). For 3D spatial interpolation we implemented a special R-package with help of the library “CGAL” [41], since available R implementations of 3D spatial interpolation have very poor performance.

For trilinear interpolation the R-package oce [49] is used.

For 3D-SSA, we use the Rssa R-package [50]. This implementation is highly effective, since it uses the approaches from [31, 37, 51].

## 4. Example for Spherical-Cap Nuclear Pattern

Here we work through an application of the 3D-SSA procedure on the close-to-spherical zebrafish egg. Nuclear coordinates are taken from the default MatchIT [4] example. The “MatchIT” tool was run with the default dataset and parameters, producing files with automatically detected nuclei. Processing is on the file “gsc_ntla_wt_t008_ch01.csv” containing nuclear coordinates for the cells expressing the* gsc* (“goosecoid”) gene at the “late shield” developmental stage.

In this data, nuclei are located in a thick layer on a sphere. Each nucleus is marked by “1” or “0,” for the gene expressing or not, respectively. For this data set,* gsc*-expressing nuclei (“1”-s) are located within a small spherical cap. We extend the analyzed area to include all “1”-nuclei, plus a surrounding ring of nuclei.

We consider two artificial (intensity) expression patterns for these nuclei and show that shaped 3D-SSA can soundly extract both types of patterns. The first kind of pattern is two-exponential, analogous to 2D* Drosophila* patterns analyzed in [35]. The second type of pattern approximates the on/off (“1”/“0”) expression values by a smooth bell-shaped pattern.

We first present results for both examples and explain the choice of parameters and pattern components. We then show how the method can be used to estimate the model of noise, after extracting the pattern. In the considered examples, we add Gaussian noise to patterns; however, it is not essential, since the SSA-family of methods is stable with respect to noise distribution and possible weak dependencies.

### 4.1. Two-Exponential Expression Pattern

Let us construct the expression values as

(3) sψ,φ,de2φ+9ψ+2d+2e−2φ+13ψ+d,

(4) vψ,φ,d=sψ,φ,d+ɛ·sψ,φ,d,

where *ψ*, *φ* are relative spherical coordinates, *d* is layer depth (*d* ∈ [0, 1]), and *ɛ* is white Gaussian noise with standard deviation 0.35. This pattern, which we call CAP-2EXP, is the sum of two exponentials plus multiplicative noise. Note that the pattern depends on three coordinates and therefore it is varied in three directions. Moderate noise levels here and in the other considered simulated examples were chosen for better visual color representation of the results for the considered patterns.

**Figure 3:**
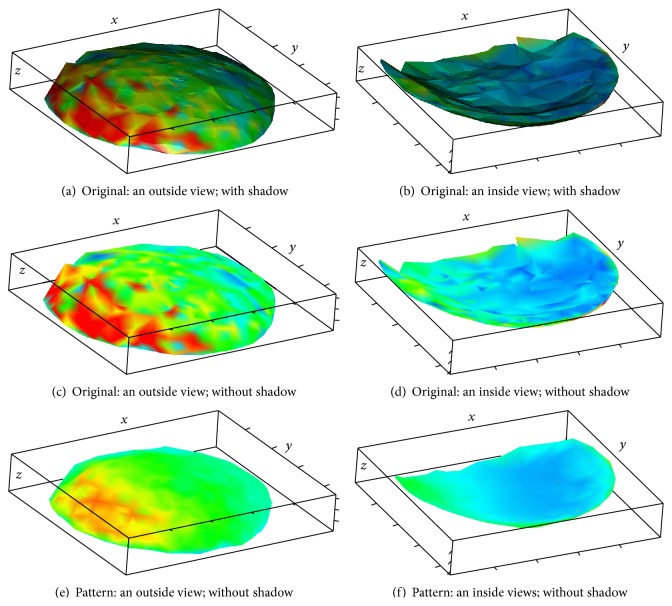
CAP-2EXP: the nuclear hulls, coloring based on original and pattern expression intensities.

Inside and outside views of the nuclear hulls are shown in Figures 3(a)–3(d), where colors correspond to expression levels, as in Figure 1. It can be seen that the coloring is variegated (nuclei of different color occupy similar positions), reflecting the presence of noise.

The method described in Section 3 produces the reconstructed pattern depicted in Figures 3(e) and 3(f). The results of noise removal can clearly be seen.

**Figure 4:**
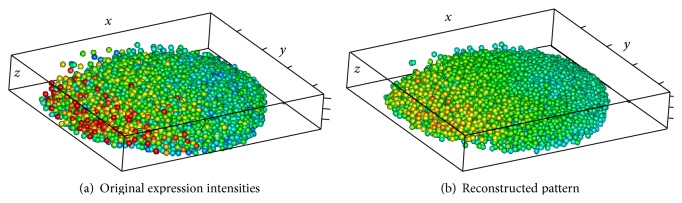
CAP-2EXP: nuclei colored; an outside view.

**Figure 5:**
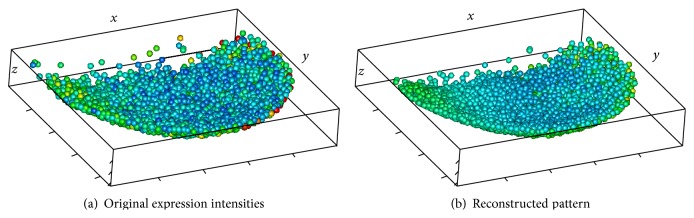
CAP-2EXP: nuclei colored; an inside view.

For better visual representation, the nuclei themselves can be depicted; see Figures 4 and 5. As before, the color of a nucleus reflects the expression level in the same scale as in Figure 1. The expression pattern can be clearly seen in the denoised data. Difference between the inside and the outside views demonstrates pattern dynamic in depth direction.

After pattern extraction, the noise model can be estimated (see Section 3.6). In (3), multiplicative noise with *α* = 1 was simulated. Applying the Park method provides an estimate of $\hat{\alpha }=1.073$, recovering the multiplicative character of the generated noise and demonstrating how the process can distinguish between, for example, additive, Poissonian, and multiplicative noise in datasets.

### 4.2. Bell-Shaped Expression Pattern

We now test the shaped 3D-SSA process on a different intensity pattern, using the same nuclear data, gsc_ntla_wt_t008_ch01.csv. We construct a bell-shaped intensity pattern (referred to as CAP-BELL) as an approximation of 0-1 expression indicators in the data, plus white Gaussian noise with standard deviation 0.25. The approximation was performed by twofold 3D-SSA smoothing and therefore it cannot be expressed by an equation.

**Figure 6:**
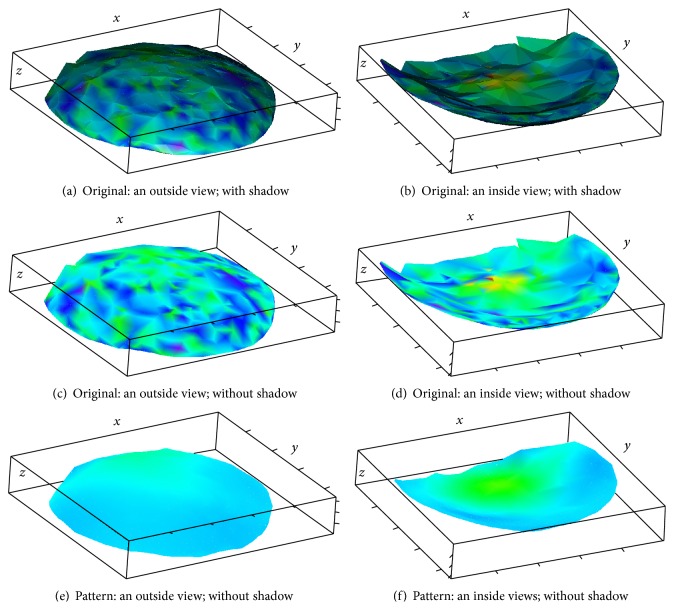
CAP-BELL: the nuclear hulls, coloring based on original and pattern expression intensities.

Inside and outside views on the nuclear hulls are shown in Figures 6(a)–6(d), where colors correspond to expression levels, in the topographic scale described in the caption of Figure 1. It can be seen that the coloring is variegated, signifying the presence of noise.

The method described in Section 3 produces the pattern depicted in Figures 6(e) and 6(f); it can be seen that the 3D-SSA procedure has removed the original noise.

**Figure 7:**
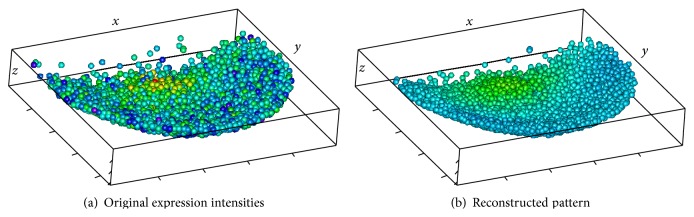
CAP-BELL: nuclei colored; an inside view.

For better visual representation, the nuclei themselves can be depicted; see Figure 7. As before, the color of a nucleus reflects the expression level in the topographic scale described in Figure 1. The pattern (trend) can clearly be seen in the denoised data.

Estimating the noise model by the Park method returns an $\hat{\alpha }=0.005$. $\hat{\alpha }$ close to zero indicates additive noise; the process has recovered the simulated Gaussian white noise.

### 4.3. The Chosen Parameters

After flattening, as described in Section 3.2, a parallelepiped with length *l* ≈ 1030, width *w* ≈ 885, and depth *d* ≈ 50. Since the shape of the flattened nuclear cloud is similar to a spherical cap, the equidistant azimuthal projection was applied.

The stepsize was chosen the same in all directions to obtain 10^6^ grid points.

For the shaped 3D-SSA algorithm described in Section 3.4, the ellipsoid window was inscribed in a parallelepiped of size *L*_1_ × *L*_2_ × *L*_3_, where the *L*_*i*_ are equal to 40% of the original image sizes. The total number of nuclei in the data file is 3595, while the chosen window covers approximately 160 nuclei on average. The number of nuclei covered by all positions of the chosen window is 3306; that is, a few side nuclei were not considered.

**Figure 8:**
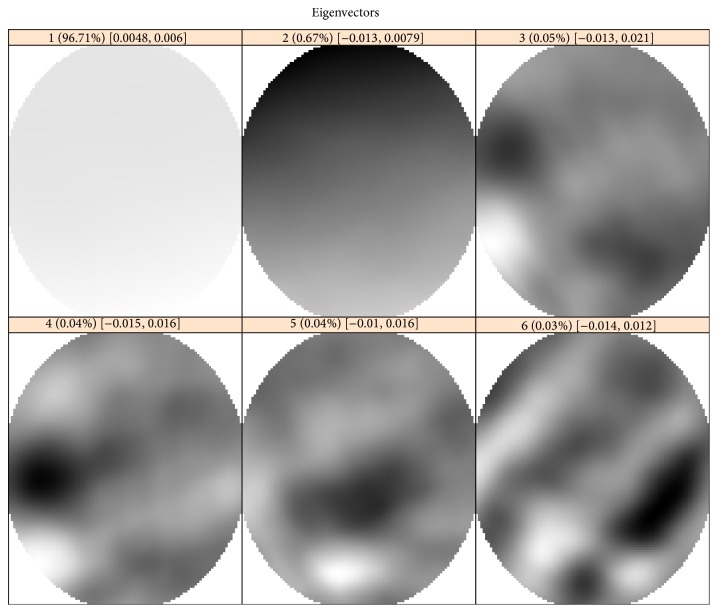
CAP-2EXP: 2D slices of 3D eigenarrays at *d* = *L*_3_/2.

To identify pattern components, let us examine 2D slices of 3D eigenarrays and elementary reconstructed components. For example, for the CAP-2EXP data (Section 4.1), Figure 8 shows slices of the six leading eigenarrays at a depth value of *L*_3_/2. The first two ellipsoidal eigenarrays are smooth and the third one has some oscillations. Therefore, we choose the first two components for pattern reconstruction.

**Figure 9:**
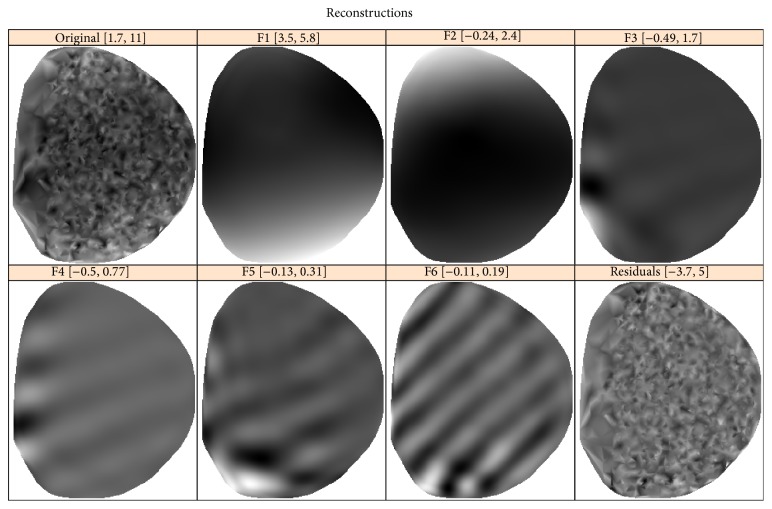
CAP-2EXP: elementary component reconstructions, slices.

The six leading elementary components, which are generated by the six eigenarrays depicted in Figure 8, together with the original and residual 2D slices are shown in Figure 9.

Figure 9 confirms the choice of the two leading components (i.e., *r* = 2) as corresponding to the pattern.

The pattern is reconstructed from the leading eigenarrays on the regular grid points, then interpolated back to flattened (irregular) nuclei, and then transformed to the original nuclear positions on the zebrafish egg.

A similar choice of pattern components can be made for the CAP-BELL data. Here, there are *r* = 3 smooth pattern components, not surprising due to the more complex form of the pattern.

The reconstruction result is quite robust to the window choice. For example, the pattern reconstruction is approximately the same if we choose a window size of 35% instead of 40% of the original size. However, for more complex patterns, smaller window sizes are preferable; see discussion regarding the choice of window in [34] (1D case) and [31] (2D case).

### 4.4. Check of Proper Pattern Extraction

Inspection of the reconstructed images clearly demonstrates the noise removal for both test patterns. However, this does not prove that we have reconstructed the whole pattern. We now show, on the CAP-2EXP example, that the pattern reconstruction is complete.

Let us consider 2D slices of the reconstructed values on the regular grid of flattened nuclei fixing the depth. The vertical axis will represent expression values (color mapped in, e.g., Figure 3) and the horizontal axes correspond to *ϕ*, *ψ* nuclear or grid-point positional coordinates.

**Figure 10:**
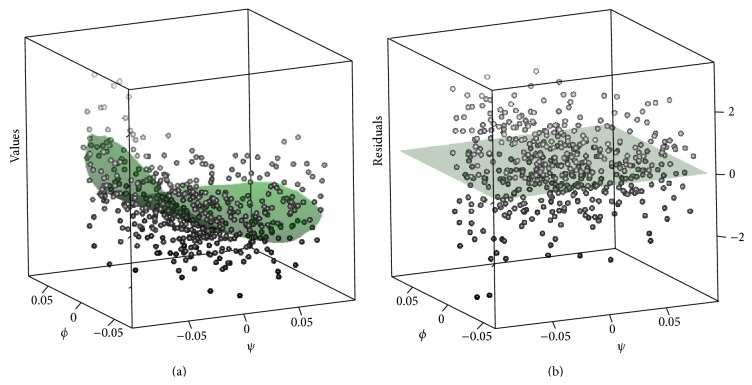
CAP-2EXP pattern: 2D slice with expression values, depth *d* = 82.5%; (a) the surface depicts the reconstructed pattern (in the regular grid points), the points show expression values in individual nuclei from a ±10% layer; (b) nuclear residuals from the reconstruction are scattered evenly around the zero plane.

Since the nuclei may not be located exactly on the slice, we consider nuclei from the layer plus-minus 10% to each side. The extracted pattern on the nuclear slice is depicted by a solid surface, with nuclear expression values shown as individual dots; see Figure 10(a). In Figure 10(b), the residuals are obtained by subtraction of the pattern from the nuclear values. The even scatter of the residuals around the zero “*xy*”-plane indicates a good fit of the reconstruction to the data.

**Figure 11:**
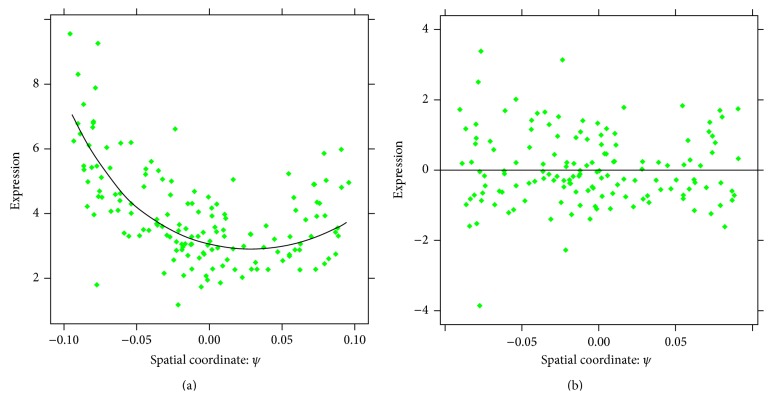
CAP-2EXP pattern: 1D slice at 82.5% depth (*d*) and 50% width (*φ*). (a) Pattern on the grid (curve) and original values on the nuclei (points). (b) Residuals between the reconstruction and the nuclear data values.

For a more refined analysis, we can construct 1D slices. For example, fixing the depth at 82.5% and the width (*φ*) at 50%, we consider the two-exponential pattern for nuclei from a thin layer of (±10% around this depth and width). The extracted pattern on the nuclear slice is depicted by a solid line.  Figure 11 confirms the quality of the reconstruction, with the residuals between the data and the reconstruction evenly spaced around the zero plane. Note that, for estimation of the noise model, the choice of 1, 2, 3, or 4 leading components has little effect on the results.

### 4.5. Graphical Analysis of Residual Model

To check the correctness of the model determined by the Park method, we can do a visual inspection of plots of the residuals divided by the trend in a degree $\tilde{\alpha }$ on the vertical against the trend on the horizontal. In such plots, the normalized residuals $r_{i}^{\left(\tilde{\alpha }\right)}$ will be most evenly distributed around *y* = 0 (homoscedastic) for $\tilde{\alpha }$-values closest to real *α*.

**Figure 12:**
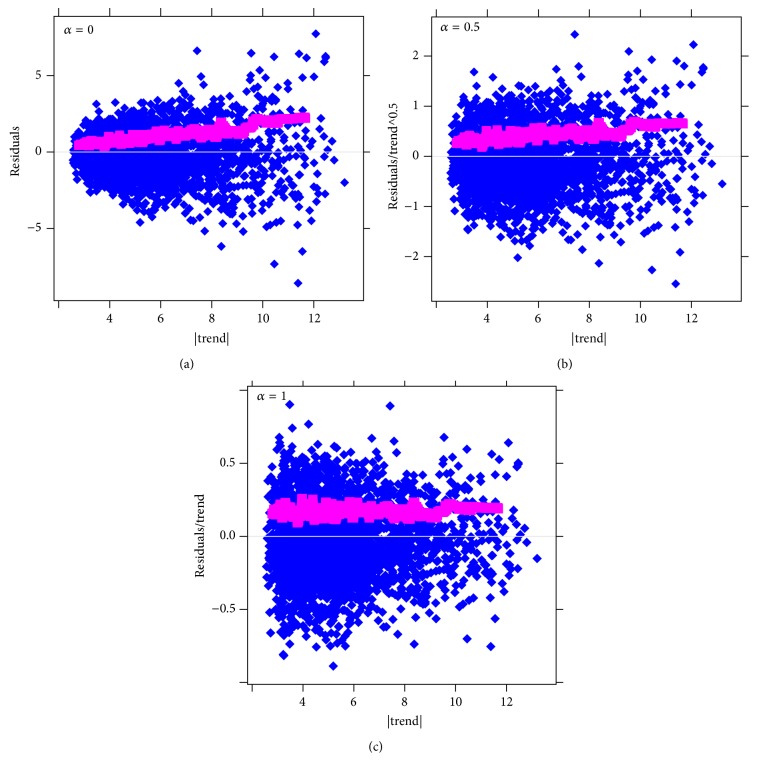
CAP-2EXP pattern, normalized residuals. (a) Test for the additive ($\tilde{\alpha }=0$) noise model. (b) Test for Poissonian noise ($\tilde{\alpha }=0.5$). (c) Test for multiplicative noise ($\tilde{\alpha }=1$). The homoscedasticity of the residuals on (c) indicates that the noise is multiplicative, confirming the noise model used in simulated generation of the data (3).

Figure 12 shows such plots for $\tilde{\alpha }=0,0.5,1$.  Figure 12(c) shows the most homoscedastic residuals (also note that the moving median of the absolute residual values, magenta, is the most horizontal): this indicates that 1 is the best estimate for *α* in this case, as expected for the multiplicative noise model in the CAP-2EXP simulated data (3).

## 5. Example for Equatorial Strip Nuclear Pattern

In this section, we analyze a qualitatively different expression pattern at a different stage of zebrafish development. We use the file 120618a_Localn0t1.emb from the “AtlasIT” tool [4], labelled for the* ntla* gene, 6.3 hours after fertilization. Expression is labelled in binary, as above. These data belong not to a particular embryo, but to a common 3D template in the atlas. Either individual embryos or common templates can be used, since we are using experimental nuclear positions, not intensities.

The nuclei cover a half-sphere, but the gene-expressing nuclei (those labelled “1”) are located in a narrow strip near the equator. Equatorial strips can be nonconvex and of varying width; hence we need more sophisticated processing than above.

As before, we will start with pattern extraction and then explain the specifics of the procedure and the parameter values.

### 5.1. Two-Exponential Expression Pattern

We model the pattern of gene expression along the strip (STRIP-2EXP) as

(5) $$\begin{array}{c} v\left(\;x,y,z\;\right)=e^{\left(y-50\right)/250}+6e^{\left(50-y\right)/500}+ɛ, \end{array}$$

where *ɛ* is white Gaussian noise with standard deviation 0.25.

**Figure 13:**
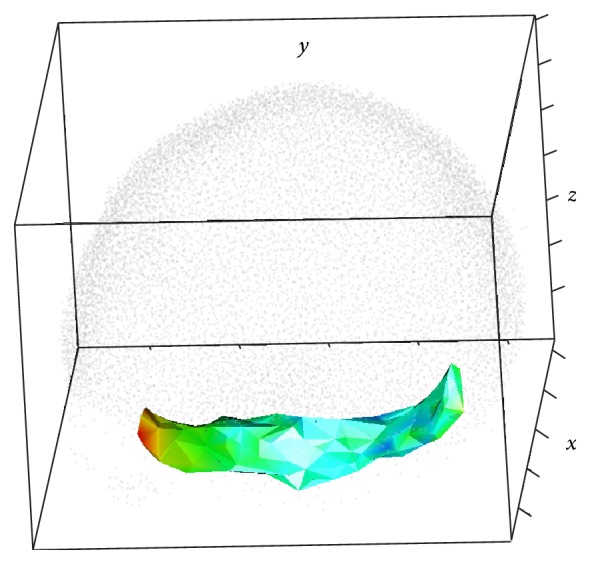
STRIP-2EXP pattern (3), nuclear hull, coloring based on the original simulated data; outside view, with shadow.

**Figure 14:**
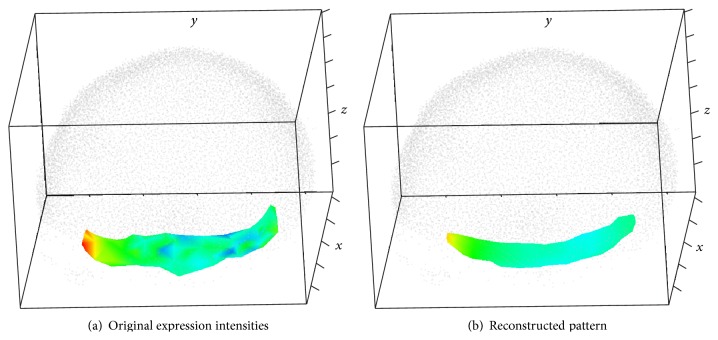
STRIP-2EXP: colored nuclear hull; outside views; without shadow.

Outside views on the nuclei hull are shown in Figures 13 and 14(a), with colors representing expression levels in the scale defined in Figure 1. Color variegation indicates the presence of noise; also note that the data area is not convex.

The method described in Section 3 produces the pattern depicted in Figure 14(b). The noise removal can clearly be seen. The area of the reconstructed pattern is visibly smaller than the original one, since the ellipsoidal window can not reach narrow parts of the original area.

**Figure 15:**
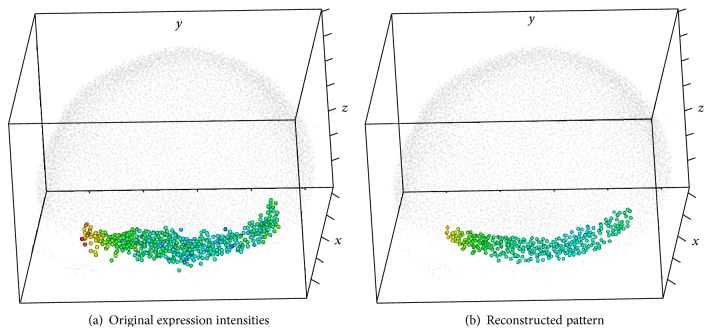
STRIP-2EXP: colored nuclei; outside views; without shadow.

Figures 15(a) and 15(b) compare the reconstruction pattern to the original expression data, plotted on separate nuclei.

### 5.2. Parameter Selection and Result Validation

After the flattening procedure described in Section 3.2, we apply the method “alphashape” [45] with a parameter equal to 5000 for construction of a nonconvex hull. The bounding box has length *l* ≈ 450, width *w* ≈ 85, and depth *d* ≈ 30. For the nuclear cloud in a strip near the equator, we applied the equidistant cylindrical projection.

As in Section 4, equal step size in all directions was used to obtain 10^6^ grid points.

For the shaped 3D-SSA algorithm described in Section 3.4, the ellipsoid window was inscribed in a parallelepiped of size *L*_1_ × *L*_2_ × *L*_3_, where the *L*_*i*_ are 35% of the original image sizes. The total number of nuclei is equal to 492; the chosen window covers approximately 19 nuclei on average. The number of nuclei covered by all positions of the chosen window is 360; that is, one quarter of the nuclei were not considered.

**Figure 16:**
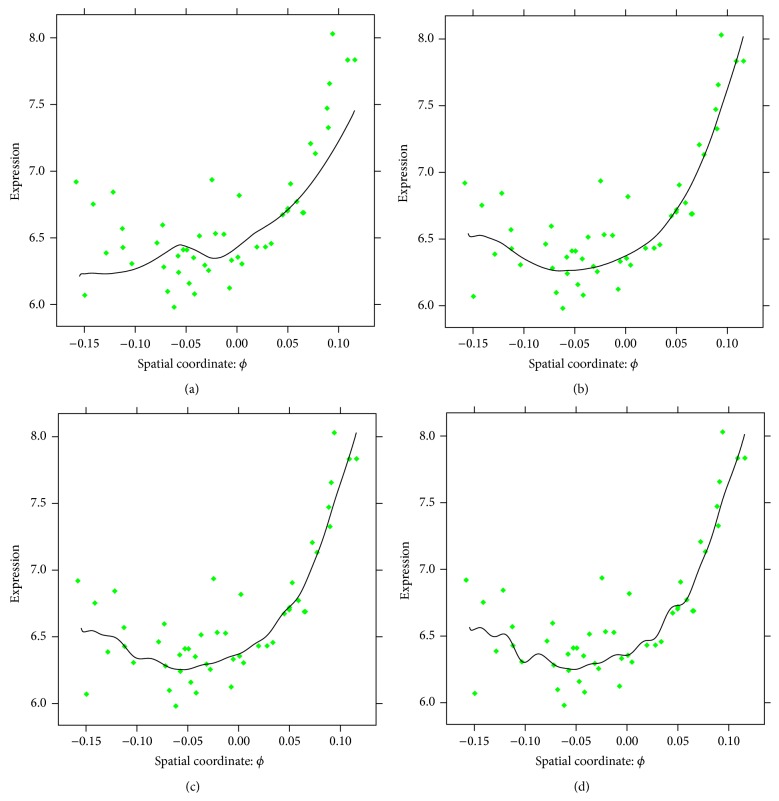
STRIP-2EXP: 1D slices for 50% depth (*d*) and width (*ψ*), signal reconstructed by 1 (a), 2 (b), 3 (c), and 4 (d) components.

As in Section 4, analysis of elementary components helps to detect the pattern (trend) components. 1D slices (Figure 16) confirm that the pattern is described by the two smooth leading components, while the third and fourth components can reasonably be assigned to the residuals.

Due to the small number of nuclei in the equatorial strips, we do not estimate the noise model.

## 6. Discussion and Conclusions

What is the biological impact of extracting denoised (and regularized) data for 3D gene expression? In particular, what can be learned from 3D noise filtering and noise analysis?

### 6.1. Gene Expression Variability and Noise

As with 1D expression data (profiles) and 2D data (expression surfaces) [26, 31, 35, 52], 3D expression data demonstrates pattern variability from embryo to embryo, as well as expression noise between neighboring nuclei in the same embryo.

*Gene Expression Variability.* As observed by Castro-González and colleagues [4], 3D gene expression volumes can vary significantly from embryo to embryo at the same developmental stage. This can manifest as additional rows of cells around the core gene-expressing domain ([4, Figure S16]). 

**Figure 17:**
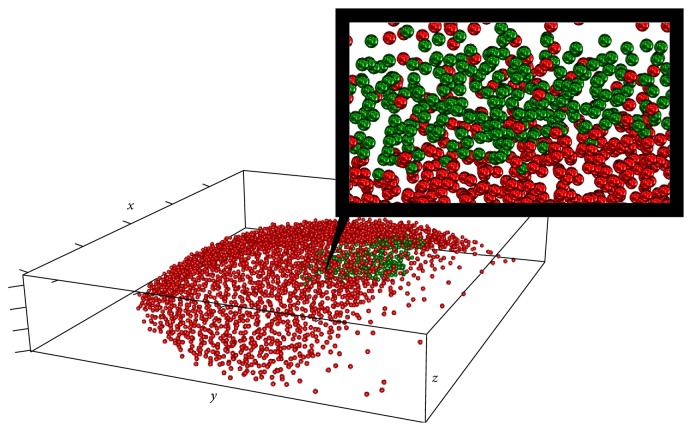
The distribution of nuclei (red and green dots) in a shield stage early zebrafish embryo, with green dots corresponding to the nuclei expressing gene* ntla*. Higher magnification in the insert shows mixing of green and red (*ntla* off) nuclei along the boundary of the expression domain.

*Gene Expression Noise.* Within-embryo expression noises (differences between neighboring nuclei) are observable both in qualitative and quantitative representations of the gene expression data [4].  Figure 17 shows qualitative-type noise in the mixing of expressing (green) and nonexpressing (red) nuclei along a pattern boundary.

Binary (on/off) data allows us to observe some degree of noise, but only as “ruggedness” at the expression domain boundary. With quantitative data, such as in Figure S10 from [4], noise between neighboring nuclei can be observed throughout the expression domain.

### 6.2. Prospects for GRN Modeling

The work in [4] studied 9 genes which are components of the axial mesendoderm gene regulatory network (GRN) proposed by Chan et al. [53]. In particular, [53] proposed that a subset of 3 of these genes (*Foxh1*,* sox32*, and* gsc*) forms the regulatory mechanism for territorial exclusion during endomesoderm specification: the common activator* Foxh1* activates both the endoderm transcription factor* sox32* and the mesoderm transcription factor* gsc*, and* sox32* then turns off expression of the mesoderm activator (*gsc*) in endoderm-lineage cells [53].

The next step in a systems biology approach would be to develop a mathematical model for such a GRN motif which could generate the proper gene expression patterns in the biological tissue geometries. As we have shown in our test cases, the 3D-SSA approach described here can extract such expression patterns from complex biological geometries. In addition, correspondence between the mathematical model and the expression data can be made at an intermediate stage of the procedure, for example, on the flattened regular grid, which may be much easier than operating in the original coordinates.

### 6.3. Prospects to Analyze 3D Expression Noise

Mathematical formulations of GRNs are most commonly developed as deterministic models, and, as discussed above, 3D-SSA can provide clear pattern trends for matching and developing such models. Increasingly, as a next step, models are also being formulated for gene expression noise, such stochastic modelling can, by treating the variation as well as the mean of the expression, serve to greatly reduce the potential model dynamics and parameters, as well as to characterize how biophysical mechanisms modulate noise (e.g., [27]). SSA is well-established and has been used effectively for extracting and analyzing noise from expression data in 1D (subsection below) and 2D (below) for a number of years. This has allowed for estimations of the model of noise (*α* values), as well as providing data against which to test stochastic model output (e.g., [26]).

*Extraction of 1D Expression Profiles and Noise.* While 1D expression data (spatial profiles) have been publicly available for more than a decade (“FlyEx” [54] and “BID BDTNP” [2, 19] Web resources), few publications have been devoted to decomposition of the data into biological trend and noise components. Wu and coauthors [55], extracted the “Bicoid” (*Bcd*) transcription factor noise to compare to stochastic simulations but relied on parameterizing the* Bcd* profile as an exponential. However, a nonparametric SSA-related approach showed a much closer fit for the sum of two exponentials [35]. SSA was also used to show that* Bcd* noise follows an additive model for the considered dataset [36], while the multiplicative model was correct for the* hb* factor noise [26]. On the* Drosophila* gene* hb*, SSA showed that a major component of the total biological noise was “texture noise,” from the precellular compartmentalization of the embryo [52]. 

*2D Expression Surfaces and Expression Noise.* There have been less studies on expression data in 2D (expression surfaces). He and coauthors [56] studied nucleus-to-nucleus* Bcd* differences and found a signal-dependent rise in variability for lower* Bcd* intensities ([56, Figure S1]). Both cited publications on the Bcd noise [55, 56] used 2D data, that is, stripes from the confocal images with several hundred nuclei, and those stripes do have not only a length, but a width too. Further analysis of the inherently 2D data as 1D, ignoring the width, can introduce substantial and unavoidable source of noise and bias conclusions on the biological noise. However, application of nonparametric 2D-SSA directly to 2D data takes into account a spatial distribution of expression intensities and therefore diminishes a possible bias. In particular, on both live and fixed marker techniques for* Bcd*, 2D-SSA indicated a signal-independence of the noise [36]. This suggests that* Bcd*, one of the most studied developmental genes, deserves further analytical study. 

*3D Expression Signals and Expression Noise.* The groundwork laid in the 1D and 2D applications of SSA indicates that the present extension to 3D can be an effective way to process data from expression data in complex geometries.

For developmental patterning in geometries which are truly 3D, such as the zebrafish embryo, or which become 3D (as with* Drosophila* after gastrulation), 3D-SSA is promising for extracting trends and noise from expression data. It could, for example, serve as a critical tool for developing a data-driven model for the* Foxh1*,* sox32*, and* gsc* motif, both deterministic and stochastic.

In conclusion, our work with 3D-SSA resolves several problems in the study of 3D gene expression. These are (i) representation of the data in a geometrically “flattened” form suitable for further analysis; (ii) interpolation of the data to a regular grid; (iii) decomposition of the data into signal and noise; and (iv) addressing some of the issues (slicing) for visualizing and evaluating results with four-dimensional data (3D + gene expression intensity). We present 3D-SSA as an adaptable and powerful technique for processing and analyzing the growing amount of gene expression data from truly 3D developmental events. Since there is an extension of 3D-SSA to 4D-SSA and even for general* n*D-SSA, the SSA family of methods is potentially able to analyze data of different spatial and temporal nature.
